# Human Development V: Biochemistry Unable to Explain the Emergence of Biological Form (Morphogenesis) and Therefore a New Principle as Source of Biological Information is Needed

**DOI:** 10.1100/tsw.2006.234

**Published:** 2006-10-23

**Authors:** Søren Ventegodt, Tyge Dahl Hermansen, Trine Flensborg-Madsen, Maj Lyck Nielsen, Birgitte Clausen, Joav Merrick

**Affiliations:** ^1^ Quality of Life Research Center, Teglgårdstræde 4-8, DK-1452 Copenhagen K, Denmark; ^2^ Research Clinic for Holistic Medicine, Copenhagen, Denmark; ^3^ Nordic School of Holistic Medicine, Copenhagen, Denmark; ^4^ Scandinavian Foundation for Holistic Medicine, Sandvika, Norway; ^5^ Interuniversity College, Graz, Austria; ^6^ Vejlby Lokalcenter, Vejlby, Denmark; ^7^ Zusman Child Development Center, Soroka University Medical Center, Ben Gurion, University of the Negev, Beer-Sheva, Israel; ^8^ National Institute of Child Health and Human Development, Jerusalem, Israel; ^9^ Office of the Medical Director, Division for Mental Retardation, Ministry of Social Affairs, Jerusalem, Israel

**Keywords:** holistic biology, theoretical biology, biochemistry, gradients, mathematic modeling, Denmark

## Abstract

Today's biomedicine builds on the conviction that biochemistry can explain the creation of the body, its anatomy and physiology. Unfortunately there are still deep mysteries strangely “fighting back” when we try to define and understand the organism and its creation in the ontogenesis as emerging from biochemistry. In analysing this from a theoretical perspective using a mathematical model focusing on the noise in complex chemical systems we argue that evolving biological structure cannot in principle be a product of chemistry. In this paper we go through the chemical gradient model and argue that this is not able to explain the ontogenesis. We discuss the used gradients as information carriers in chemical self-organizing systems and argue that by use of the “Turing structures” we are only able to modelling the mostly simple biological systems. The bio-chemical model is only able to model simple organization but not to explain the complexity of biological phenomena. We conclude that we seemingly have presented a formal proof (a NO-GO theorem) that the self-organizing chemical systems that are using chemical gradients are not able to explain complex biological matters as the ontogenesis. We need a fundamentally new, information-carrying principle to understand biological information and biological order.

## INTRODUCTION

A way to understanding the cell is looking at what the cell definitely is not. One popular metaphor in understanding the nature of the living cell has been the cybernetic "controlling machine", which besides just simply producing proteins and when needed exposing these on the surface of the cell membrane i.e. to express its membership of a specific type of tissue, also have the unique capability of organizing the information for the ontogenesis of the tissue into complete functional structures through genetic control. In this paper, we argue that the simple gradient model proposed for morphogenesis of proteostome animal lines does not find its parallel with the deuterostome lines; chemical gradients do not explain even the simplest biological functions in vertebrates. The theoretical reason for this is quite obvious: (1) chemical gradients do not work as information donors in self-organizing biological systems, as the law of diffusion causes a distinct lack of precision through this process, making the iteration needed for development of complex animals impossible; using the theoretically possible reaction-diffusion structures as basis for morphogenesis does not work for the same reason; (2) using this model on embryos does not reveal the predicted disturbance of the development; (3) the developmental stages of, for example, the giant tadpole, seem to exclude the possibility of diffusion (chemical signaling) as informational backbone in developing systems. Therefore, chemical gradients and signaling molecules in general fail to explain complex biological functions. In this paper, we discuss the involvement of the cell in the ontogenetic process and the failure of the chemical gradient model to explain the ontogenesis.

### Gradients Fail to Explain the Ontogenesis

*Gradients as information promoters of self-governmental systems:* As apparent, hormones and trophic substances can be used by biological systems to determine absolute measures as, for example, agents that decide sizes of head and body of the cnidarian hydroid *Hydra vulgaris*[1] and the decision of the amount of motor neurons through NGF (nerve growth factor)[2]. But as implied by the problems described below, the information necessary for this organization hardly comes from a time-space network of signal molecules.

The information for cell determination in a developing embryo, generally, could be sourced from the genes. From these, the information could be spread out through a complex time-space network of signal molecules. Such systems are known from cybernetics[3], but the reliability of these involves three main problems. (1) To avoid a chaotic outcome through time, all steps (specially the first steps) in such a sequential development have to be extremely precise. But, unfortunately, the law of diffusion that normally is used to describe the roomy distribution of signaling molecules causes a distinct lack of precision in such systems[4]. (2) Using this model on embryos (on morula and blastula stages) does not disturb the development; not even after violent manipulations[4], as this model implies. Therefore, the dynamics of developing systems makes this model less reliable. (3) Tyroidea hormone generation of the metamorphosis in the giant tadpole[4] indicates that the morphogenetic hormones themselves do not transform the information to form the shape. Instate, it seems like these, through positional information, are involved in a superior control of the manifestation of the genetic information. For example, the final positional organization of a cell may be built by a sequence of positional informational items registered through time[4], but because of the huge distance in such biological systems (max 2 mm owing to the time), such stages of development also seem to exclude the possibility of diffusion.

The biological complexity arises from sequential recruitment of global organizations called reaction-diffusion structures (or “Turing structures”[5]). This caused sensation when the inclusion of Ficks diffusion-law was shown to result in roomy organization in equation systems for many autocatalytic reactions[5]. Therefore, it was proposed that such a kind of organization delivers potential information through morphogenesis. Since then, a lot of scientists have used variations of this equation to explain roomy organization in biological systems (e.g., J.D. Murray, H.G. Othmer, G.F. Oster, A. Hunding, B. Goodwin, L. Wolpert, and H. Meinhart). These researchers have also used enzyme reaction systems with excitatory feedback in the modeling of biological systems. The solution of such nonlinear differential equations could result in roomy organization of different kinds. Also “Turing analogue” models have been developed. In these, the diffusion link is replaced by another, but similar, kind of link that is due to other cell interactions as active transport and interactions through formation[6]. Also, the thermodynamic equations have been included in such calculations without any further result[7]. This activity has involved the development of “Turing structures” into a subgroup of “dissipative structures” arising from self-organization of systems ahead from equilibrium. Very few of the last-mentioned model types, but none of the first-mentioned, have yet been discovered.

### Understanding Matter, Life, and Consciousness: Making No-Go Theorems for Life as Chemical, Self-Organizing Systems

The recognition that living organisms are organized in a fractal manner on a number of levels (7,11) makes it possible to test our assumptions concerning living organisms in a fractal manner. By use of the No-Go theorem, we will here try to show the formal impossibility of well-organized multicellular life based on the ordinary recognized molecular biological assumptions concerning living organisms as self-organizing molecular systems. It seems that No-Go theorems can make it necessary to make a revision of basic assumptions that normally are granted.

It is hard to understand the informational systems of the living organism and it does not seem that quantum mechanics can explain the conditions behind the existence of the fundamental substances in living systems. The disciplines of consciousness (cognitive, religious, and philosophical disciplines) are not able to explain consciousness’s ability to create wholeness, coherence, and meaning in a chaotic world of details.

Because our consciousness is developed from the living organism, it is obvious to consider which information processing traits of the living organism are able to create the superior joined properties of the consciousness. The conditions of information in biological systems are still very poorly understood. Living organisms seem to exist by means of an information system interacting with all cells simultaneously and with the single cells individually. This information system looks to be fractal organized[8], so its structure is parallel with the fractal organization of the living organisms. But until now, the traits that link the organization of organisms on all levels is not understood. This information system could very well be the foundation of consciousness. Classically, this trait is described as the quality about the biological system of information that makes it possible to establish superior connections as, for example, size relations, despite the unstable or chaotic relations of the sublevels. An example of this is the kidney tubulus from the caterpillar of the newt. These are able to maintain the kidneys absolute size in spite of huge enlargements of the underlying level necessary for formation of the structure (the size and amount of the cells expand). Concerning polyploid and haploid (bigger and smaller) cells, a corresponding major or minor amount of cells is used for the actual structure; see Fig. 1[9]. The knowledge deduced from this has been that the biological information is linked to roomy conditions more likely than enumerations of the amount of cell proliferations and similar concepts.

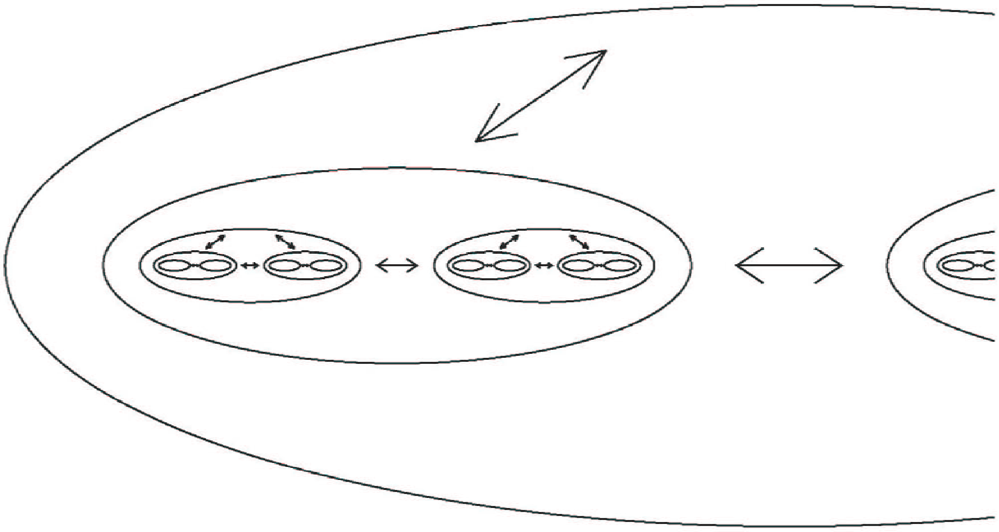

**FIGURE 1.** The fractal organization of the living organism, with parts that interact, and by this forms a number of levels. The arrows illustrate information-transmitting interactions and are generalized from the empirical findings. This illustrates how biologic systems act as a fractal kind of Chinese box containing the information-transmitting interactions between the different parts of the same level.

The classical expression for this has been "positional information" or "the biological (information) field"[4]. It has been proposed that diffusion-dissipation structures could deliver such roomy positions[7], but below we will also show that if it is theoretically accepted that the roomy structure should be able to be organized by nonlinear, chemical processes in this way, the dynamic, fractal organized information system, including the superior regulatory functions of biological systems, could not be described by this in an appropriate way.

### A No-Go Theorem for the Living as Self-Organizing, Molecular Systems

We will try to show that chemical self-organizing systems are not capable of giving the observed precision of structures concerning the superior levels. In the fractal organized system (Fig. 1), the parts interact and, thereby, give wholeness on a number of levels corresponding to a complete individual organism. We will try to show that it never can be more precise than the underlying levels. When the last-mentioned levels fluctuate in time, we think it gives even more inaccuracy. The No-Go theorem only counts for the static situation. A No-Go theorem for the (chaotic-) dynamic situation of balance is under construction.

Because the information is restricted to the DNA, the molecular biological understanding of the living organism is that signaling agents carry the roomy organization. This information is only able to escape the DNA molecule during the action of other molecules through transcription, translation, enzyme activity, etc. Therefore, we are able to project the living organism into a multidimensional room of chemical gradients as shown in Fig. 2. The living organism is represented as a lane (trajectory) in this room. The lane may never cross itself, because this would involve a collapse of a representation necessary for creating and keeping up the organism.

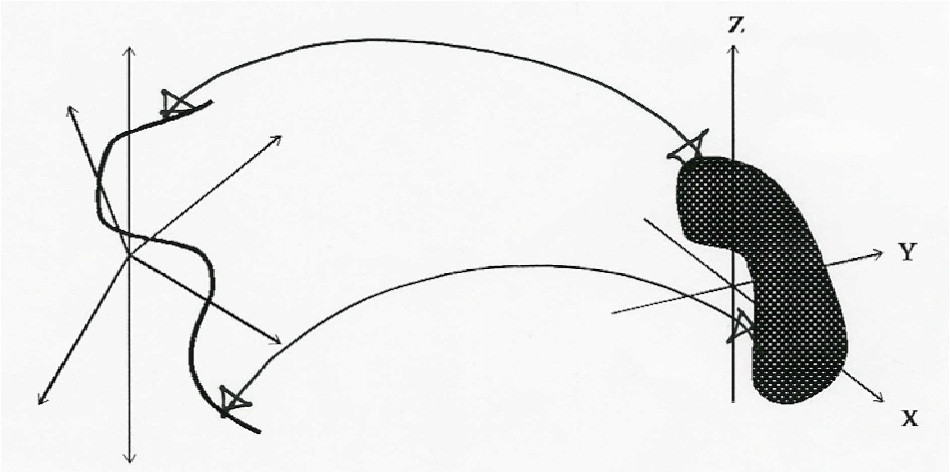

**FIGURE 2.** The living organism is projected to a multidimensional room of chemical gradients[10].

The known physics and chemistry claim that all interactions are local (chemical agents diffuse or they are transported around). The multidimensional gradient room, therefore, may have a metric that makes it possible to maintain global order because local interactions alone would involve too big an uncertainty on the superior levels and huge distances (see Fig. 3). Such a metric, however, will in itself block for the dynamics existing in a living organism because a specific structure with a specific size (e.g., kidney tubulus, a human leg of a specific length) is necessary to base it on the underlying metric in its construction. The underlying level, therefore, may not fluctuate more than the superior level. The example involving the amount of cells in the newt tubulus is a model example.

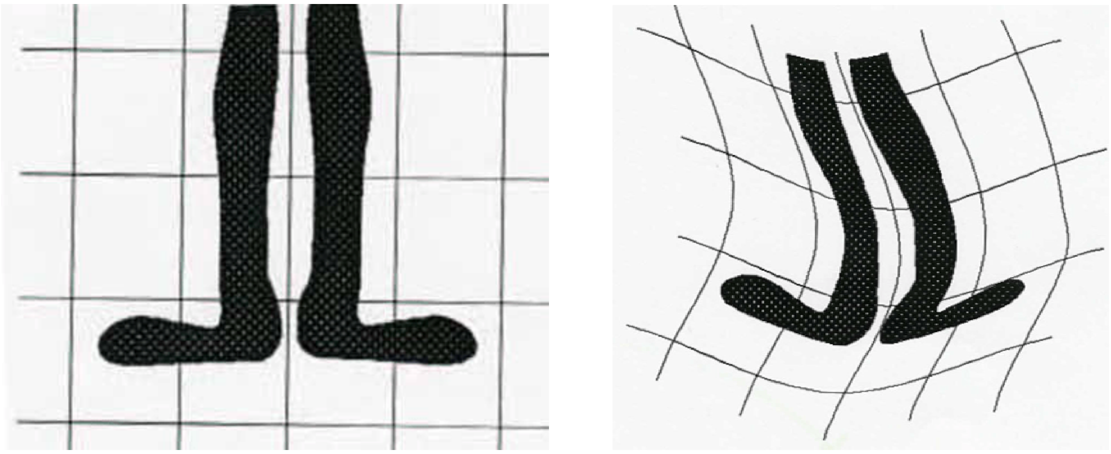

**FIGURE 3.** Without a firm metric (right), the organisms form and other high-level qualities will not be able to be established or maintained in a system only based on local interactions[10].

Conclusively, a matrix in the multidimensional room of chemical gradients having the necessary firmness will fixate the system dynamic in a degree struggling against the biological empery. A looser metric will allow the observed dynamic, but will not be able to function as “bag-bone” for exact organization on higher levels and over huge distances. An example of the consequence of this is that a human’s leg length of about 1 m, that from leg to leg of the same person, is very exact within a few per mille, demands a metric that makes the presence of legs of the same length with all people, and this is not the case. Thereby, it seems to be excluded (through static terms) that the known laws of molecular biology can explain the biological informational conditions. This means that biological information systems could be based on constant changes of a kind unknown until now, or that an unknown physical-chemical law exists, including organizing qualities, as the living organism has been able to use.

### No-Go Theorem for Phenomenologically Observed Precision in Biological Systems at Macroscopic Scales

Examples are given of large-scale precision in biological systems. The following example is a transcription from Bennett et al.[10]: It is inexplicable given that the known fluctuations in the number of underlying fractal structures exceed the tolerances to which macroscopic precision is observed. As a concrete example, we have in mind the precision ($5^{o}/_{oo}$) to which the two legs of an average human being have the same length.

The assertion that this macroscopic precision is inexplicable is made relative to a model for biological organization utilizing the inverse of a mapping *f* from the 3-dimensional geometrical space $\mathbf{R}^3$ in which the organism is embedded to a very high-dimensional space **K** spanned by the concentrations of all the chemical components of the organism.

If the map $f$: $\mathbf{R}^3 \rightarrow \mathbf{K}$ is to have a chance of leading to a “blueprint” (residing in the space **K**) from which a biological organism can be constructed in $\mathbf{R}^3$, the mapping *f* must certainly be injective. Otherwise the mapping *f* would not have an inverse. Not having injectivity would correspond to an ambiguity in position in $\mathbf{R}^3$ when attempting to use the “blueprint” to fabricate the organism in $\mathbf{R}^3$. A potentially interesting speculation is that such ambiguities could be an important factor in certain pathological conditions; for example, an ambiguity as to where a biological structure starts and where it stops could lead to uncontrolled replication.

However, more than the injectivity of the map *f* is required. In order for *f* to have an inverse; we must have a way of providing a restriction in the high-dimensional space **K** such that this restriction coincides with the image of the mapping *f*. A possible restriction that could be used comes from the reasonable assumption that the observed stability of biological organization must necessarily be supported by complicated neg-entropic biochemical processes (necessary, for example, to avoid “demise by diffusion”). Such complicated biochemical processes must be expected to place severe constraints on relationships between the concentrations of chemical components. Processes that are stabile with regard to small violations of such constraints must be assumed for biological processes [such stability is seen in "buffered" systems for example]; maybe the more fashionable way to talk about this is to say “buffering by commodity conservation as a way of enforcing the presence of two or more phases, the transition between which are first order (in order to give “gap” needed for fine-tuning)”] - could be expected to define a manifold embedded in **K**. Such a manifold would correspond to a. “limit cycle” (“attractor”, “strange attractor"??) dictated by such (stabile) biochemical processes necessary for the maintenance of biological organization.

Let **M** designate the manifold (assumed differentiable) embedded in **K** that corresponds to this “limit cycle” and thereby to the restriction necessary to ensure the invertibility of the map *f*. Because our hoped for biological “blueprint” is forced to reside on the manifold **M** (i.e., the limit cycle), we can adopt as a metric on **M** that induced in tangent spaces to **M** by the directional derivatives along various chemical component directions. Thus endowed with a metric, the surface **M** becomes a geometric surface and one might then hope that the inverse mapping $f^{-1}$: $\mathbf{K}|_{\mathbf{M}} \rightarrow \mathbf{R}^3$ could be used as a “blueprint” from which the biological system in $\mathbf{R}^3$ could be constructed.

Consider now the trajectories $R(t)$ and $L(t)$ in $\mathbf{R}^3$ along, respectively, a right and left leg that transform into each other under (discrete) symmetry operations in $\mathbf{R}^3$ (please notice that trajectories *R(t)* and *L(t)* are traditionally marked with arrows above the letter). It is further assumed that these trajectories start and end at macroscopically corresponding points on the two legs in accordance with having two legs of the same length. For example, we can think of trajectories $R(t)$ and $L(t)$ that transform under reflections as $\hat{A}R(t) = L(t)$ (t is a parameter; $\hat{A}$ is the lateral reflection operator in the middle plane of the human body). We would expect that these trajectories $R(t)$ and $L(t)$ would be mapped by $f$ into trajectories $f(R(t))$ and $f(L(t))$ in **M** that were "parallel" or close together in some sense. This would ensure that integration of the metric (induced in **M**) along these trajectories in **M** would be equal (in accordance with the assumption of (symmetric) trajectories in $\mathbf{R}^3$ along left and right legs of the same length). The reason for expecting this equality

$$\int_{f(R(t))} \sqrt{g}\, ds = \int_{f(L(t))} \sqrt{g}\, ds \qquad (1)$$

is that the integrals along trajectories $f(R(t))$ and $f(L(t))$ that are "close together" in **M** "feel" the essentially same variation in the metric.

Now the following question must be addressed: Given the magnitude of known fluctuations in the numbers of self-similar structures (e.g., cells) that make up a leg, we must ask if our candidate for a biological "blueprint" residing in **M** can accommodate these fluctuations and still function as a map into $\mathbf{R}^3$ that can lead to a right and a left leg that have the same length to within a tolerance known to be less than that that would correspond to the fluctuations in the number of underlying cellular building blocks. The essence of this No-Go theorem is the demonstration that this *is* not possible without come sort of "fine tuning" in the form of additional assumptions.

The idea behind the "proof" is roughly as follows. The trajectories $f(R(t))$ and $f(L(t))$ in **M** (corresponding, respectively, to the trajectories $R(t)$ and $L(t)$ in $\mathbf{R}^3$ described above) can be expected to be spirals. The periodicity (forget about the "pitch" of the spiral for a moment) comes from the fractal-like (cellular) nature repeatedly encountered on a trajectory (in $\mathbf{R}^3$) through a biological material. On the other hand, parameters in **M** that reflect the number of cells encountered along a trajectory obviously cannot be periodic; hence, the "pitch" of the spiral trajectory in **M** corresponding to the trajectory in $\mathbf{R}^3$ along the length of a leg. The number of cells encountered along a trajectory can be expected to be topologically encoded in **M** as the winding number of the spiral trajectory. Now the naturally occurring (i.e., known) fluctuation in the number of cells encountered in the trajectory along the right leg and the left leg would show up as different winding numbers for these two trajectories in **M**. However, we know that these two trajectories $f(R(t))$ and $f(L(t))$ can be expected to be roughly parallel ("close together") according to the discussion above. The essence of our No-Go is the impossibility of having two parallel spirals (in **M**) with different winding numbers: the known relative fluctuation in the number of underlying fractal structures making up a macroscopic organism leads to different winding numbers for the trajectories $f(R(t))$ and $f(L(t))$, thereby precluding that these can be parallel in **M** as required by Eg. (1). This mismatch in our hoped-for biological "blueprint" prevents the construction in $\mathbf{R}^3$ of a right and left leg that have the same length to within the phenomenologically observed precision.

### DISCUSSION

Simple gradient models do not seem to be able to explain ontogenesis[2]. With the existing knowledge about DNA and genes, it is rational to think that information in biological systems is directed from the genomes, through a chemical arrangement. Transport of information in such systems has been explained through gradient models, trying to state how the information is conveyed from genes to shape — some examples are the axon excrescence through a gradient of NGF, and morphogenesis through a gradient of sticky-molecules as N-CAM and NG-CAM[11]. In the simplest versions — as the "French flag model"[12,13] — a gradient of a signal molecule ("morphogene") is formed through a "source" and a "drain". Cells in between these could be imagined to have concentration-sensitive receptors with thresholds that can only be reached in some areas of the system, but this is still hypothetical. The idea is that activation of the receptor should control the specific gene expression, but even the simplest forms of the gradient model[2] have shown to be unfunctional without the assumption that the threshold variegates locally. By assuming this, the cells themselves could have the information to create complex biological patterns. The gradient model, therefore, is able to explain simple biological functions, but not capable of explaining complex matters as morphogenesis by itself.

The existing knowledge concerning protein processing makes it logical to think that information in biological systems is distributed from the genes. We do not think this could explain the information for ontogenesis alone. For example, the information responsible for the organization of agents deciding the sizes of body and head of *H. vulgaris*, or the decision of the amount of motor neurons through NGF, are hardly directed from a time-space network of signal molecules. Therefore, it does not seem reasonable that simple gradient models should be able to explain the complete information pattern necessary to organize ontogenesis. Even the simplest forms of the gradient model have shown to be unfunctional. However, by assuming that the thresholds vary locally, the cells could get the information to create the complex biological patterns by themselves. This, on the other hand, is only an assumption. Even when we use the “Turing structures”, we are only able to make models of the most simple biological systems. Therefore, this model is not able to explain the complexity of biological phenomena. This means that the chemical gradient model is able to explain simple biological functions, but not able to explain complex matters, such as morphogenesis.

The idea that sequential recruitment of global organizations is able to activate morphogenesis is a good idea, but the question is if the reaction-diffusion systems really are able to create the necessary “prepatterns”. Since the middle 1970s, the “Turing structures” have been criticized because of problems like: (1) experimentally, none have succeeded in proving these structures as a roomy organization of a three-dimensional system, (2) theory and practice have shown that it is impossible to model complex, reproducible, reaction-diffusion structures (Axel Hunding, personal communication). If a structure has to be defined as roomy, using a solution (length of structure/wavelength) of only $5 \times 5 \times 5$, the reproducibility will vanish completely. The border for creation of roomy organization seems to be divided into about ten compartments (Axel Hunding, personal communication). This means that by use of the “Turing structures”, we only are able to model the most simple biological systems. Therefore, this model is only able to model simple organizations as, for instance, in the fruit fly *Drosophila*, egg polarization, gastrulation, and compartmentalization of the eggs, but it is not able to explain the complexity of biological phenomena.

The point of view of contemporary molecular biology is that information to the organization of living beings is tied to genetic activity[4], but the paradox of the C-value (C = complexity)[2] and the macroevolution[15] makes this less likely. According to the C-value paradox, the beings of high evolutionary levels are more complex, but, on the other hand, they have not much more DNA than simple (including single-celled) organisms[4]. The macroevolution, the evolution of the shape of living beings, seems to be independent of the microevolution, the evolution of molecular structures. For example, the DNA of human beings and chimpanzees are 99% identical. This is the same difference as seen between twin-species of mammalians and fruit flies, respectively[4]. Based on this, the No-Go theorem makes it possible that something else stands behind the biological organization.

It can be argued that both compartimentation and segmentation in the Drosophila are very characteristic for the complexity of biological phenomenon, while they do not enable us to understand the inner part of the biological frame. Is it allowed to ask if there will be any necessity to model the whole biological scene? The Nobel-prize-authors of Drosophila-segmentation characteristics won their experimental explanations (DNA-types) from the biological phenomenon from the midst between simple organisation on one side and complex organisations on the other side. Very fine done! Still chemistry seems not to be able to solve the problem of the emerging controlled morphological complexity in biological systems.

### CONCLUSION

The presented No-Go theorem makes it likely that something else than biochemistry is behind the biological information capable of organizing morphogenesis.

### ACKNOWLEDGMENTS

These studies were supported by grants from IMK Almene Fond. Our research in quality of life has been approved by the Copenhagen Scientific Ethical Committee under number (KF)V.100.2123/91.
